# Supplementary material for: An Automated Hardware-in-Loop Testbed for Evaluating Hemorrhagic Shock Resuscitation Controllers
Source: Bioengineering (Basel). 2022 Aug 7;9(8):373. doi: 10.3390/bioengineering9080373 (PMC9405047; doi:10.3390/bioengineering9080373)
Supplement: Supplementary file 1 [file bioengineering-09-00373-s001.zip › bioengineering-1827628-SI.pdf]

Supplementary Information

Supplementary Table S1: Summary of each controller configuration performance at two bleed rates for all controller performance criteria during testing scenario #1. This scenario represents an initial hemorrhage to 40 mmHg MAP with an ongoing hemorrhage clotting with time. Fluid resuscitation was with whole blood in this scenario.

	**Testing Scenario # 1**			
	**Low Bleed Rate**		**High Bleed Rate**	
	120s Sampling	*5s Sampling*	*120s Sampling*	*5s Sampling*
Effective(%)	76.72	75.38	72.95	71.53
MDPE(%)	-1.50	-0.99	-1.39	-1.05
MDAPE(%)	2.84	0.99	3.46	1.05
Overshoot(%)	6.33	0.69	4.08	0.91
Divergence(%/hr)	-28.36	-3.30	34.20	-3.43
Efficiency(min)	4.08	4.58	6.08	5.75
Wobble(%)	1.48	0.14	1.63	0.15
MeanInf(ml/min)	34.79	25.28	36.70	28.01
Volume Efficiency	2.24	3.83	2.03	2.75
Area Above Target (min)	0.16	0.00	0.21	0.00
Area Below Target (min)	-1.25	-1.27	-1.46	-1.53
Area Below Target @Rise Time (min)	-0.98	-1.03	-1.20	-1.27

Supplementary Table S2: Summary of each controller configuration performance at two bleed rates for all controller performance criteria during testing scenario #2. This scenario represents a rapid loss of blood due to mimic tourniquet failure and whole blood resuscitation without an active hemorrhage.

	**Testing Scenario # 2**			
	**Low Bleed Rate**		**High Bleed Rate**	
	120s Sampling	*5s Sampling*	*120s Sampling*	*5s Sampling*
Effective(%)	76.04	83.90	84.10	83.90
MDPE(%)	-1.13	-0.96	-0.11	-0.96
MDAPE(%)	2.55	0.96	2.30	0.96
Overshoot(%)	6.84	0.70	5.22	0.63
Divergence(%/hr)	-4.36	-2.77	-1.70	-2.78
Efficiency(min)	4.42	2.42	2.83	2.42
Wobble(%)	1.52	0.13	1.78	0.12

MeanInf(ml/min)	22.73	14.56	25.00	14.56
Volume Efficiency	1.97	3.62	1.73	3.59
Area Above Target (min)	0.21	0.00	0.23	0.00
Area Below Target (min)	-1.12	-0.66	-0.59	-0.65

Supplementary Table S3: Summary of each controller configuration performance at two bleed rates for all controller performance criteria during testing scenario #3. This scenario mirrors the previous scenarios rapid loss of blood but highlights the vasopressor capabilities of the system for 10 minutes followed by whole blood resuscitation.

	**Testing Scenario # 3**			
	**Low Bleed Rate**		**High Bleed Rate**	
	120s Sampling	*5s Sampling*	*120s Sampling*	*5s Sampling*
Effective(%)	83.42	89.30	86.02	92.15
MDPE(%)	-0.16	-0.92	-1.60	-0.88
MDAPE(%)	1.95	0.92	3.16	0.88
Overshoot(%)	8.76	0.63	4.33	0.75
Divergence(%/hr)	-11.07	-2.56	-28.87	-2.93
Efficiency(min)	4.67	2.50	4.08	1.58
Wobble(%)	1.21	0.11	1.61	0.13
MeanInf(ml/min)	21.71	11.80	25.34	10.73
Volume Efficiency	1.32	3.33	1.36	2.98
Area Above Target (min)	0.37	0.00	0.36	0.00
Area Below Target (min)	-1.06	-0.76	-1.18	-0.57

Supplementary Table S4: Summary of each controller configuration performance at two bleed rates for all controller performance criteria during testing scenario #4. This scenario uses crystalloid as the infusate type after a large hemorrhage and no coagulopathy results in an ongoing hemorrhage throughout the scenario.

	**Testing Scenario # 4**			
	**Low Bleed Rate**		**High Bleed Rate**	
	120s Sampling	*5s Sampling*	*120s Sampling*	*5s Sampling*
Effective(%)	81.50	83.42	3.87	64.35

MDPE(%)	-2.08	-1.18	-8.34	-6.92
MDAPE(%)	2.08	1.18	8.34	6.92
Overshoot(%)	7.49	0.82	5.56	0.70
Divergence(%/hr)	-1.94	-4.40	0.70	3.42
Efficiency(min)	3.83	3.08	1.50	1.17
Wobble(%)	0.68	0.09	0.39	0.75
MeanInf(ml/min)	33.06	36.78	49.31	50.00
Volume Efficiency	1.33	1.47	1.14	1.14
Area Above Target (min)	0.00	0.00	0.00	0.00
Area Below Target (min)	-1.29	-1.08	-2.83	-2.30

Supplementary Table S5: Summary of each controller configuration performance at two bleed rates for all controller performance criteria during the entire testing regimen.

	**Across Entire Testing Regimen**			
	**Low Bleed Rate**		**High Bleed Rate**	
	120s Sampling	*5s Sampling*	*120s Sampling*	*5s Sampling*
Effective(%)	79.97	83.48	59.01	77.59
MDPE(%)	-1.31	-1.04	-3.58	-1.12
MDAPE(%)	2.27	1.04	4.98	1.12
Overshoot(%)	2.22	0.63	1.22	1.56
Divergence(%/hr)	-11.43	-3.26	1.08	-1.43
Efficiency(min)	4.25	3.15	3.63	2.73
Wobble(%)	1.22	0.12	1.36	0.29
MeanInf(ml/min)	27.84	22.69	34.74	27.06
Volume Efficiency	1.17	1.36	1.13	1.17
Area Above Target (min)	0.74	0.00	0.80	0.00
Area Below Target (min)	-4.71	-3.77	-6.06	-5.05
